# Concentrations of Phenolic Acids Are Differently Genetically Determined in Leaves, Flowers, and Grain of Common Buckwheat (*Fagopyrum esculentum* Moench)

**DOI:** 10.3390/plants10061142

**Published:** 2021-06-03

**Authors:** Alena Vollmannová, Janette Musilová, Judita Lidiková, Július Árvay, Marek Šnirc, Tomáš Tóth, Tatiana Bojňanská, Iveta Čičová, Ivan Kreft, Mateja Germ

**Affiliations:** 1 Faculty of Biotechnology and Food Sciences, Slovak University of Agriculture in Nitra, Tr. A. Hlinku 2, 949 76 Nitra, Slovakia; janette.musilova@uniag.sk (J.M.); judita.lidikova@uniag.sk (J.L.); julius.arvay@uniag.sk (J.Á.); marek.snirc@uniag.sk (M.Š.); tomas.toth@uniag.sk (T.T.); tatiana.bojnanska@uniag.sk (T.B.); 2 Research Institute of Plant Production, Bratislavska 2795/122, 921 01 Piestany, Slovakia; iveta.cicova@nppc.sk; 3 Nutrition Institute, Tržaška cesta 40, Sl-1000 Ljubljana, Slovenia; ivan.kreft@guest.arnes.si; 4 Biotechnical Faculty, University of Ljubljana, Jamnikarjeva 101, Sl-1000 Ljubljana, Slovenia; mateja.germ@bf.uni-lj.si

**Keywords:** *Fagopyrum esculentum*, buckwheat, leaves, flowers, grain, phenolic acids, flavonoids

## Abstract

Common buckwheat (*Fagopyrum esculentum* Moench) is a valuable source of proteins, B vitamins, manganese, tryptophan, phytochemicals with an antioxidant effect, and the natural flavonoid rutin. Due to its composition, buckwheat supports the human immune system, regulates blood cholesterol, and is suitable for patients with diabetes or celiac disease. The study aimed to compare the allocation of selected phenolic acids (neochlorogenic acid, chlorogenic acid, trans-caffeic acid, trans-*p*-coumaric acid, trans-sinapic acid, trans-ferulic acid) and flavonoids (rutin, vitexin, quercetin, kaempferol) in the leaves, flowers, and grain of buckwheat cultivars of different origin. The content of individual phenolics was determined by the HPLC-DAD method. The results confirmed the determining role of cultivar on the relative content of chlorogenic acid, trans-caffeic acid, trans-sinapic acid, vitexin, and kaempferol in buckwheat plants. A significantly negative correlation among concentrations of phenolic acids in different common buckwheat plant parts shows that there are different mechanisms of genetic influences on the concentration of phenolic substances in common buckwheat flowers, leaves, and grain. These differences should be taken into account when breeding buckwheat for a high concentration of selected phenolic substances.

## 1. Introduction

In Europe, buckwheat is a crop of importance in Slovakia, Czech Republic, Poland, Lithuania, Latvia, Ukraine, Belarus, Russia, Luxemburg, Italy, and Slovenia. In Asia, it is cultivated mainly in India, Nepal, Bhutan, China, Korea, and Japan [1,2,3,4,5]. As a consequence of changes in the environment as well as in the lifestyle of modern civilization, there is a rapid increase of so-called lifestyle diseases, food intolerances (or non-allergic food hypersensitivity), and allergies [6]. Improper nutrition as well as an improper composition of foods with excessive energetic intake; high consumption of lipids, simple sugars, and salt; and also insufficient intake of fiber, vitamins, and substances with a prophylactic value are factors contributing to this negative phenomenon [6,7,8,9]. The reintroduction of crops with an excellent chemical composition, which had a long tradition of cultivation in some regions, is one of the ways to change the eating habits with the aim to contribute to improving the health of the population. Buckwheat is one among the mentioned traditional high-quality crops [4,5,10,11,12,13].

The excellent chemical composition of buckwheat provides the possibility to use several parts of the plant, not just achenes but also flowers and leaves. Young leaves and flowers of buckwheat plant have been used both for medicinal and culinary purposes in Europe and Asia [1,2]. When used as a food, the leaves are cooked and consumed as a vegetable, or they are ground into a fine flour, which is then used in bakery products. When used medicinally, dried buckwheat leaves are utilized for herbal tea preparation or processed into supplements.

Buckwheat grains are rich in proteins, polysaccharides, dietary fiber, lipids, polyphenols, macro- and microelements [1], as well as vitamins B_1_, B_2_, and B_6_ [2], vitamin E, and trace quantities of β-carotene [3]. Protein content in buckwheat grains ranged from 12% to 19% [4]. The high quality of buckwheat protein with biological values can be explained by a high concentration of the most essential amino acids, especially lysine, tryptophan, threonine, and the sulfur-containing amino acids [5]. The composition of proteins enables buckwheat grain to be used as a non-gluten-containing food source, with applications in the prophylaxis of gastrointestinal tract diseases, mainly celiac disease [10]. Starch content in buckwheat grains varies from 59% to 70% [11], total dietary fiber represents 5% to 11% of buckwheat grains [12]. Buckwheat grains contain from 1.5% to 4% of total lipids [13]. Buckwheat is rich in macroelements, such as potassium, magnesium, calcium, and sodium as well as microelements such as iron, manganese, and zinc. In very low levels, trace elements, such as chromium or selenium in buckwheat grain are also detected [14]. Buckwheat grain contains a number of components with prophylactic value and biological activity, such as flavonoids, phenolic acids, condensed tannins, phytosterols, and fagopyrins [1,15]. Buckwheat plant parts contain fagopyrins, phototoxic phenolic substances belonging to the group of anthraquinones. Common buckwheat leaves have lower levels of fagopyrins than Tartary buckwheat (*Fagopyrum tataricum*) or cymosum buckwheat (*Fagopyrum cymosum*). Fagopyrin is found in the green parts of plants and less in grains. After steaming, the fagopyrin content in the grains decreases. Buckwheat grain and flour products are safe for consumption in normal amounts. Diets composed to a large extent of buckwheat herbs, flowers, or sprouts may cause fagopyrism [16,17,18,19]. However, fagopyrin may protect buckwheat from pathogenic fungi [20]. Buckwheat is the only grain crop containing rutin and quercetin. Other plants typically containing rutin or quercetin are herbs or trees. Rutin is contained in rue (*Ruta graveolens* L.) in leaf tissues at about 14 mg/g d.w. [21]. Holm oak (*Quercus ilex* L.) leaves typically show contents of up to about 0.034 mg/g quercetin-hexosides in d.w. [22]. For neochlorogenic acid, St. John’s wort (*Hypericum perforatum* L.), it is a typical herb with up to about 17.8 mg/g d.w. [23]. The flavonoid rutin, of which buckwheat is an excellent source, deserves special attention. Rutin has interesting effects on the human organism, it reduces high blood pressure, reduces the risk of arteriosclerosis, and has antioxidant activity [24]. The content of buckwheat phenolics can be influenced by geographic origin of grain, environmental conditions [25] as well as the growth phase of the plant, with the highest being at the end of the vegetation period [26]. In common buckwheat plants, the concentration of rutin is much higher in common buckwheat flowers in comparison to that in the grain [27,28]. It is not yet known for common buckwheat, whether there is any connection between the concentration of rutin and phenolic acids in grains, leaves, and flowers among varieties.

The aim of this study was to investigate the concentration of selected phenolic acids and flavonoids in leaves, flowers, and grains of common buckwheat with different origin. A further aim was to investigate whether a possible correlation exists in the concentration of studied metabolites in flowers, leaves, and grains, or on the other side, whether there are differences in metabolite spectra among plants of diverse genetic backgrounds.

## 2. Results and Discussion

### 2.1. Flavonoids and Phenolic Acids in Buckwheat Leaves

The leaves of buckwheat are characterized by a range of pharmacological effects through antioxidant mechanisms due their phenolic constituents [29]. The determined content of selected phenolic acids in the leaves of investigated buckwheat cultivars are given in Table 1.

Results (Supplementary Figure S1) indicate that the contents of phenolic acids in the leaves are very variable. A significantly higher content in the Kasho-2 cultivar was observed for the neochlorogenic acid, while a statistically lower content was recorded in the Pulawska cultivar. Significantly higher contents of chlorogenic acid were recorded in the Kasho-2 (*p* < 0.01) and Pennline 10 (*p* < 0.05) cultivars. Significantly lower content of chlorogenic acid was recorded in the Pulawska (*p* < 0.05) and Soldier Pond (*p* < 0.01) cultivars. In the case of trans-caffeic acid, significantly higher contents were recorded in the Pyra (*p* < 0.01) and Hrusowska (*p* < 0.05) cultivars; on the other hand, significantly lower contents were detected in the FAG 120/82 (*p* < 0.05) and Soldier Pond (*p* < 0.01) cultivars. Significantly lower levels of trans-coumaric acid were recorded in the Pyra (*p* < 0.01) and Pulawska (*p* < 0.05) cultivars, while significantly higher levels were recorded in Kasho-2 (*p* < 0.05) and Hrusowska (*p* < 0.01) cultivars. The cultivars Soldier Pond (*p* < 0.01) and FAG 120/82 (*p* < 0.05) are characterized by a significantly lower content of trans-ferulic acid, while Pyra (*p* < 0.05) and Pennline 10 (*p* < 0.01) have a significantly higher content of trans-ferulic acid. FAG 120/82 and Soldier Pond have a statistically significantly higher content of trans-sinapic acid (*p* < 0.05; *p* < 0.001).

Žvikas et al. [30] determined in buckwheat leaves 0.21–2.80 mg/g d.w. of neochlorogenic acid and 0.02–0.13 mg/g of chlorogenic acid. Our results are similar to those of Žvikas et al. [30]. Significantly lower values of chlorogenic acid content are reported in different buckwheat cultivars from China, Japan, and Korea 58 and 72 days after sowing [31]. The content of chlorogenic acid in the leaves of two common buckwheat cultivars 58 (72) days after sowing was 0.02 mg/g d.w. (ND) and 0.06 mg/g d.w. (0.09 mg/kg d.w.), respectively. On the other hand, Sytar [32] reported a higher content of chlorogenic acid (3.27 mg/g d.w.) as well as a higher content of trans-ferulic acid (29.03 mg/g d.w.) in the leaves of the buckwheat cultivar Rubra, compared to our results.

The laves of buckwheat have been considered as one of the most useful sources of rutin. Buckwheat leaves contain also other flavonoids with interesting properties, of which the selected ones were analyzed (Table 2).

Significant differences in the content of flavonoids in the leaves are shown in Supplementary Figure S2. Higher content of rutin was observed in the case of the cultivars Kasho-2 (*p* < 0.01) and Pennline 10 (*p* < 0.05). On the other hand, significantly lower content of rutin was observed in Pulawska (*p* < 0.05). A significantly higher content of quercetin was recorded in Pennline 10 (*p* < 0.05), while the cultivar Kasho-2 had a significantly lower content of quercetin (*p* < 0.05). In the case of kaempferol, higher contents were observed in the Hrusowska (*p* < 0.05) and Soldier Pond (*p* < 0.001) cultivars. The cultivars Kasho-2, Pulawska, and Soldier Pond were characterized by a significantly lower content of vitexin (*p* < 0.05; *p* < 0.05; *p* < 0.05); on the contrary, a significantly higher content of vitexin was established in the cultivars Pyra (*p* < 0.01) and Pennline 10 (*p* < 0.05).

As it was stated, the vegetative parts, particularly the leaves and flowers, are among the best-known sources of rutin. Hence, functional foods originated from buckwheat leaves are common, although the scope of such applications is limited by phototoxicity associated with the fagopyrin composition. Rutin content in buckwheat leaves reaches up to 34.17 mg/g d.w. [33]. Dražić et al. [34] reported that buckwheat leaves grown in Eastern European countries under different growing conditions have been shown to contain rutin in the range of between 21.70 and 34.30 mg/g d.w. According to the authors, the rutin content is affected not only by the environmental conditions of the growing location but also by the meteorological conditions of the growing year. Our results are in agreement with these data. Rutin contents in the leaves of European buckwheat cultivars vary in the interval 17.74 mg/g d.w. (cv. Pulawska, Poland) and 28.21 mg/g d.w. (cv. Pyra, Czech Republic); while in buckwheat cultivars from Japan (cv. Kasho-2) and USA (cvs. Soldier Pond and Pennline 10), the determined rutin content in leaves was 31.07 and 29.28 mg/g d.w. (29.67 mg/g d.w.), respectively. Zielińska et al. [35] determined a significantly higher rutin content (82 mg/g d.w.) in buckwheat leaves. Similarly, Žvikas et al. [30] reported higher values of rutin content in buckwheat leaves (31.7–66.7 mg/g d.w.) but also comparable values of quercetin content (0.21–0.55 mg/g d.w.) to our results. The contents of vitexin, rutin, and quercetin in buckwheat leaves of two cultivars 58 (72) days after sowing determined by Seo et al. [31] were 0.16 mg/g d.w. (ND) and 0.23 mg/g d.w. (0.03 mg/g d.w.), 11.1 mg/g d.w. (16.7 mg/g d.w.) and 15.9 mg/g d.w. (32.3 mg/g d.w.), 0.07 mg/g d.w. (0.19 mg/g d.w.) and 0.12 mg/g d.w. and 0.09 mg/g d.w. (respectively). These data are similar to our results. Dziadek et al. [36] investigated content of phenolic compounds in extracts and infusions of buckwheat leaves. They found that both extracts and infusions were richest in ferulic acid, while the extracts contained more polyphenols and were characterized by higher antioxidant activity than infusions. Besides phenolic compounds, buckwheat leaves and stems accumulate a remarkable amount of leucine [37], which could be used to develop new functional foods with a positive effect on the human health.

### 2.2. Flavonoids and Phenolic Acids in Flowers

Buckwheat is well known for its profuse flowering; it is attractive to insects and suitable as an ornamental plant. The buckwheat flowers are useful in infusion mixture preparation and contribute to improving the quality of honey, due to content of biologically valuable compounds. Among 90 studied Polish samples of honey, the strongest antioxidant activity was found for buckwheat honey [38]. During the harvesting period, grains (mature as well as immature grains) and a few flowers coexist at the same time on the plant [39]. In Table 3, the determined selected phenolic acids contents are given.

The average content of neochlorogenic acid in the flowers of the investigated buckwheat cultivars is 0.162 mg/g d.w., while the lowest one was determined in cv. Ballada (Russia) and the highest one in cv. Pyra (Czech Republic). Our results are in agreement to data reported by Žvikas et al. [30], who determined 0.05–0.32 mg/g d.w. of neochlorogenic acid in buckwheat flowers. However, at the same time, these authors determined in buckwheat flowers a higher content of chlorogenic acid (1.9–5.4 mg/g d.w.) compared to our values. On the other hand, Sytar [32] reported a lower content of chlorogenic acid and a higher content of trans-ferulic acid in buckwheat flowers (0.031 and 0.317 mg/g d.w., respectively) compared to results. In the other study, Sytar et al. [40] determined in the flowers of buckwheat cv. Rubra a higher content of chlorogenic acid (1.52 mg/g d.w.) and even 20.12 mg/g d.w. of trans-ferulic acid. Significantly higher values of chlorogenic acid content were reported by Seo et al. [31]. These authors measured 58 and 72 days after sowing, in buckwheat flowers, 7.18–9.27 and 6.0–6.5 mg/g d.w. of chlorogenic acid, respectively. These results confirm the fact, that cultivar is among the most important factors influencing the content of phenolic acids in buckwheat plant.

The graphic representation of the content of phenolic acids in the flowers is in Supplementary Figure S3. No significant difference in neochlorogenic acid content compared to the median were confirmed. However, in the case of chlorogenic acid, significantly higher levels were recorded in the Kasho-2 (*p* < 0.01) and FAG 120/82 (*p* < 0.05) cultivars. The Pulawska had a significantly lower content of chlorogenic acid compared to other cultivars (*p* < 0.01). In the case of trans-caffeic acid, a significantly higher content was observed in the Pyra (*p* < 0.01) and FAG 120/82 (*p* < 0.05) cultivars, and a significantly lower content of trans-caffeic acid was recorded in the Pulawska (*p* < 0.05) and Soldier Pond (*p* < 0.01) cultivars. The cultivars Pyra and Soldier Pond had a statistically significantly higher content of trans-coumaric acid compared to other cultivars (*p* < 0.05; *p* < 0.01). On the other hand, the Ballada cultivar had a significantly lower content of trans-coumaric acid (*p* < 0.01). The Kasho-2 (*p* < 0.05) and FAG 120/82 (*p* < 0.01) cultivars had significantly higher levels of trans-ferulic acid. The FAG 120/82 and Soldier Pond cultivars had significantly higher trans-sinapic acid (*p* < 0.05; *p* < 0.01) contents, while the Pulawska (*p* < 0.05), Pennline 10 (*p* < 0.05), and Ballada (*p* < 0.05) cultivars had significantly lower trans-sinapic acid contents.

Buckwheat is the only field crop that contains rutin, of which the majority is synthesized in the flowers. Rutin contents in the flowers of investigated buckwheat cultivars (Table 4) vary in the interval 7.410 mg/g d.w. (cv. Pulawska, Pulawy, Poland)–20.847 mg/g d.w. (Soldier Pond, ME, USA).

Results (Supplementary Figure S4) display significant differences in the content of flavonoids in flowers. Significantly higher values of rutin and quercetin were recorded in the Kasho-2 (*p* < 0.01; *p* < 0.05) and Soldier Pond (*p* < 0.05; *p* < 0.01) cultivars. The Ballada and Pulawska cultivars had significantly lower contents of rutin and quercetin (*p* < 0.05). A significantly higher content of kaempferol was found in the Soldier Pond cultivar (*p* < 0.001). In the case of vitexin, a significantly higher content was recorded in the Pyra cultivar (*p* < 0.05) and a significantly lower content in the Soldier Pond cultivar (*p* < 0.01).

Žvikas et al. [30] determined a significantly higher rutin content (49.5–81.2 mg/g d.w.) but comparable quercetin content (0.18–0.41 mg/g d.w.) in the flowers of 15 common buckwheat cultivars compared to our values. Similarly, Zielińska et al. [35] reported 78 mg/g d.w. of rutin in buckwheat flowers. Seo et al. [31] measured rutin and quercetin contents in buckwheat flowers 58 (72) days after sowing, obtaining the following values: 33.2–39 mg/g d.w. (33.7–47.8 mg/g d.w.) and 1.0–2.5 mg/g d.w. (0.93–1.45 mg/g d.w.), respectively. Their values for quercetin content are consistent with ours. Literature data on rutin content could reflect the impact of ecological factors on the concentration of rutin in buckwheat flowers [2]. In comparing buckwheat samples of different origin, grown in original places [41], it is not possible to establish whether the differences among samples are due to genetic factors or are just the result of specific conditions of growing and environment. This is the first study where that experiment was performed by growing cultivars originating from different countries (Czech, Japan, Germany, Poland, USA, Russia) in the same environmental and growing conditions for the determination of the mentioned phenolic acids along with flavonoids. In this investigation, a huge difference in rutin concentration was obtained from Polish Pulawska (7.4 mg/g d.w.) to Japanese Kasho (21.2 mg/g d.w.). Similarly, a huge difference among flowers of samples was obtained in the concentration of quercetin from Polish Pulawska (about 0.09.4 mg/g d.w.) to USA sample Soldier Pond (1.68 mg/g d.w.).

### 2.3. Flavonoids and Phenolic Acids in Grains

Phenolic acids are one of the two main classes of phenolics in buckwheat grain (Table 5). The Ballada cultivar is characterized by significantly higher contents of phenolic acids (neochlorogenic acid, trans-caffeic acid, trans-coumaric acid, trans-ferulic acid, trans-sinapic acid) and significantly lower contents of chlorogenic acid. Significantly lower levels of neochlorogenic acid, chlorogenic acid, trans-caffeic acid, and trans-ferulic acid were recorded in the Pulawska cultivar. Significantly higher levels of neochlorogenic acid and chlorogenic acid (Supplementary Figure S5) were recorded for the Soldier Pond cultivar.

The content of neochlorogenic acid in the grains of investigated buckwheat cultivars varies in the interval 0.074 mg/g d.w. (cv. FAG 120/82, Germany)–0.216 mg/g d.w. (cv. Ballada, Russia). These results are higher compared to those obtained by Kiprovski et al. [41], who reported 0.001–0.006 mg/g d.w. These authors also determined a lower content of chlorogenic acid (0.003–0.013 mg/g d.w.) in buckwheat grains compared to values obtained in the present research. A similar amount of chlorogenic acid (0.008 mg/g d.w.) to that in the present study was assessed by Zhang et al. [42]. Among the determined phenolic acids, neochlorogenic acid and chlorogenic acid are most abundant in buckwheat grains. Dziadek et al. [43] confirmed that phenolic compounds are accumulated especially in the grain hulls of buckwheat, while dehulled buckwheat grains are the poorest source of polyphenols.

Flavonoids are the second main class of phenolics present in the grains of buckwheat (Table 6). Statistically significant differences in flavonoid content in the grains are shown in Supplementary Figure S6. Statistically significant higher contents of rutin, quercetin, and kaempferol were recorded in the Ballada cultivar (*p* < 0.01). On the other hand, the Ballada cultivar was the only one of the analyzed cultivars with a statistically significantly lower vitexin content (*p* < 0.01). In the case of kaempferol, significantly lower contents were observed in the Pulawska (*p* < 0.05), Soldier Pond (*p* < 0.05), and Pennline 10 (*p* < 0.05) cultivars. The Pennline 10 cultivar also has a significantly lower quercetin content (*p* < 0.01). A significantly lower content of rutin was recorded in the Pulawska (*p* < 0.01) and Pyra (*p* < 0.05) cultivars.

The determined content of the dominant flavonoid rutin in the grains of investigated buckwheat cultivars varies in the interval 2.791 mg/g d.w. (cv. Pulawska, Poland)–13.326 mg/g d.w. (cv. Ballada, Russia). These values are higher compared to those obtained by Zhang et al. [42], who determined in buckwheat grains 0.131 mg/g d.w. of rutin. Even lower values (0.062 mg/g d.w.) were presented by Liu et al. [44]. In contrast, Zielińska et al. [35] determined a higher rutin content in the grains of common buckwheat (16 mg/g d.w.) than was determined in our samples. Our results agree with those obtained by Zhu et al. [45], who determined the rutin content in the grains of six buckwheat samples from China in the range 1.29–6.29 mg/g d.w. Anyway, these authors determined a higher kaempferol content (0.101–0.140 mg/g d.w.) compared to our values. On the other hand, Zhang et al. [42] determined a significantly lower kaempferol content (0.001 mg/g d.w.) in comparison to our values. Our values of vitexin are in the interval 0.010 mg/g d.w. (cv. Ballada, Russia)–0.212 mg/g d.w. (cv. Kasho-2, Japan). These values are comparable with the results of Kiprovski et al. [41], who reported 0.003–0.033 mg/g d.w. of vitexin in buckwheat grains. Our values of quercetin content are consistent with those obtained by Liu et al. [44] as well as Mikulajová [46], who reported in buckwheat grains 0.027 mg/g d.w. and 0.002–0.027 mg/g d.w. of quercetin (respectively). In contrast, Keriene et al. [47] determined a significantly lower quercetin content (0.003–0.007 mg/g d.w.) compared to our results. The chemical composition of buckwheat grains could be improved using biostimulants or microorganisms. Witkowicz et al. [48,49] reported studies focused on the influence of biological control agents and plant growth promoters on the chemical composition and antioxidant activity of buckwheat sprouts. They confirmed that the antioxidant potential of buckwheat sprouts is essentially due to the predominant hydrophilic fraction of antioxidants.

Based on the average values of individual phenolics contents from all investigated buckwheat cultivars, we can create the following order of occurrence: neochlorogenic acid: leaves > flowers > grains; chlorogenic acid: flowers > leaves > grains; trans-caffeic acid: leaves > flowers > grains; trans-coumaric acid: leaves > flowers > grains; trans-sinapic acid: flowers > leaves > grains; trans-ferulic acid: leaves > flowers > grains; rutin: leaves > flowers > grains; vitexin: leaves > flowers > grains; quercetin: leaves > flowers > grains; kaempferol: flowers > leaves > grains.

To statistically evaluate the influence of cultivar on the content of valuable components, the differences between buckwheat cultivars (related to the whole plants) were tested. No significant differences in the contents of trans-ferulic and trans-coumaric acids between the tested cultivars were observed. In the case of trans-caffeic acid, a significantly lower content in the Pulawska cultivar was observed. On the other hand, a significantly higher content of trans-caffeic acid was observed in the Pyra cultivar. The cultivar Soldier Pond is characterized by a significantly higher content of trans-sinapic acid and a significantly lower content of trans-caffeic acid. A significantly lower content of trans-sinapic acid was observed in Pennline 10. On the other hand, the FAG 120/80 cultivar had a significantly higher content of trans-sinapic acid (Figure S1). No statistically significant differences between the tested cultivars in the neochlorogenic acid content were observed. The Kasho-2 cultivar had a significantly higher content of chlorogenic acid. On the other hand, the Pulawska cultivar had a significantly lower content of chlorogenic acid (Figure S2). 

In the case of rutin and quercetin, no significant differences between tested cultivars were observed. The kaempferol content was variable. The cultivars Pulawska and Pennline 10 had a significantly lower content of kaempferol. Statistically higher values of kaempferol were recorded for the Hrusowska and Soldier Pond cultivars. The Soldier Pond cultivar also had a significantly lower content of vitexin. In contrast, a significantly higher content of vitexin was observed for the Pyra cultivar (Figure S3).

### 2.4. Correlation between Flavonoid and Phenolic Acids Concentrations in Leaves, Flowers, and Grains

The results of the correlation analysis between the tested variables are shown in the Supplementary Figure S7. Several positive and negative relationships between the tested parameters were established. A positive relationship was confirmed between the content of neochlorogenic acid and values of chlorogenic acid (r = 0.59), trans-caffeic acid (r = 0.39), trans-coumaric acid (r = 0.81), trans-ferulic acid (r = 0.77), rutin (r = 0.69), and quercetin (r = 0.63). An inverse relationship was observed between neochlorogenic acid and vitexin (r = −0.32). An inverse relationship was also observed between chlorogenic acid and kaempferol (r = −0.49), kaempferol and trans-caffeic acid (r = −0.48), kaempferol and vitexin (r = −0.40), vitexin and trans-coumaric acid (r = −0.45), trans-caffeic acid and trans-sinapic acid (r = −0.3), and vitexin and quercetin (r = −0.21). A positive relationship was confirmed between chlorogenic acid and the remaining analytes except trans-sinapic acid, where the relationship was not significant. A positive relationship was confirmed between trans-caffeic acid and trans-ferulic acid (r = 0.6), rutin (r = 0.37), vitexin (r = 0.38), and quercetin (r = 0.23). Strong positive relationships were observed between trans-coumaric acid and trans-ferulic acid (r = 0.67), rutin (r = 0.70), and quercetin (r = 0.75). Weak positive relationships were confirmed between kaempferol and trans-sinapic acid (r = 0.29) and kaempferol and trans-coumaric acid (r = 0.20). Furthermore, strong positive relationships were observed between trans-ferulic acid and rutin (r = 0.67), trans-ferulic acid and quercetin (r = 0.57), and rutin and quercetin (r = 0.79).

A statistically significant positive relationship in the concentration of metabolites was confirmed between the leaves and flowers in the contents of chlorogenic acid (r = 0.580), neochlorogenic acid (r = 0.365), trans-caffeic acid (r = 0.521), and trans-sinapic acid (r = 0.779). The relationship between leaves and grains and grains and flowers in any of the tested acids was not confirmed (Supplementary Figure S8). Supplementary Figure S9 shows the relationships between leaves, flowers, and grains for the flavonoids tested. A positive relationship was observed between leaves and flowers for kaempferol (r = 0.756), rutin (r = 0.529), and vitexin (r = 0.385). The inverse relationship between grains and flowers in the case of kaempferol was confirmed (r = −0.419). No statistically significant relationship between leaves and grain were found in any of the flavonoids tested.

A significantly negative correlation among concentrations of metabolites in the studied common buckwheat material shows that there are different mechanisms of genetic influences on the concentration of phenolic substances in common buckwheat flowers, leaves, and grains. To reveal, how the mechanisms perform, it is necessary to ask the simplified general exploratory question by changing one genetic factor or one environment at a time [50]. These differences should be taken into account when breeding buckwheat for a high concentration of selected phenolic substances and in the selection of cultivars for diverse nutritional and pharmaceutical purposes.

## 3. Material and Methods

### 3.1. Plant Material

In this study, eight cultivars of common buckwheat (*Fagopyrum esculentum* Moench) of different origins were used (Table 7).

Plants of all investigated cultivars were cultivated in the same locality at the Research Institute of Plant Production in Piestany, Slovak Republic, under the same environmental an agrochemical conditions. The latitude and longitude of the experimental field were 48°35′08″ N and 17°48′56″ E. The soil type of the locality, Piestany, is degraded chernozem and is characterized by a good K content (284.4 mg/kg), a middle Ca content (4977.6 mg/kg), a very high Mg content (403.4 mg/kg), a low P content (43.5 mg/kg), a high humus content (3.33%), and a neutral soil reaction (pH/KCl 6.9). The annual average temperature of this locality is 9.2 °C, with an average annual rainfall of 593 mm, and the altitude is 161 m a.s.l.

The in-field sowing date was 23 April, the harvest date was in the interval 10–28 August (after 93–115 days to the maturity of buckwheat plants of each cultivar). For the material preparation, five individual plants were used. Only standard NPK fertilization (N—nitrogen, P—phosphorus, K—potassium), namely granulated mineral fertilizer, N:P:K in the relation 4:12:20, in an amount of 50 g/m^2^ was used for the achievement of a favorable soil macroelement content. No herbicides were applied to the buckwheat during the growth of the plants. The hulled buckwheat grains, leaves, and flowers were manually separated, dried at 105 °C to the constant weight (WTC Binder, Tuttlingen, Germany), and powdered (Fritsch Pulverisette, Idar-Oberstein, Germany).

### 3.2. Extract Preparation

For the extract preparation, 2 g of the homogenized dried plant samples were used, which were extracted with 20 mL 80% aqueous methanol (*v*/*v*) at laboratory temperature for 8 h usin a horizontal shaker (250 rpm) Unimax 2010 (Heidolph Instruments, GmbH, Schwabach, Germany). The extract was filtered through Munktell No. 390 paper (Munktell & Filtrak GmbH, Bärenstein, Germany) and stored in closed 20 mL PE vial tubes.

### 3.3. HPLC-DAD Determination of Selected Phenolic Contents

All solvents and chemicals (including analytical standards) were of analytical or HPLC grade bought from Sigma-Aldrich (St. Louis, MO, USA). Prior to HPLC analysis, the extracts were filtered through a syringe filter, Q-Max (0.22 µm, 25 mm, PVDF) (Frisenette ApS, Knebel, Denmark). The content of individual phenolics was determined using Agilent 1260 Infinity HPLC (Agilent Technologies GmbH, Wäldbronn, Germany) with a quaternary solvent manager coupled with a degasser (Cat. No. G1311B), sampler manager (Cat. No. G1329B), column manager (Cat. No. G1316A), and diode array detector (Cat. No. G1315C) as previously reported by Lukšič et al. [51] and Germ et al. [52]. All HPLC analyses were performed on a Purospher^®^ reverse phase C18 column (250 mm × 4 mm × 5 µm) (Merck KGaA, Darmstadt, Germany). The mobile phase consisted of gradient acetonitrile with phosphoric acid (pH = 3.5) (A) and 0.1% (*v*/*v*) phosphoric acid in deionized H_2_O (B). The gradient elution was as follows: 0–1 min isocratic elution (20% of A), 1–5 min linear gradient elution (20–25% of A), 5–15 min (25–30% of A), and 15–25 min (30–40% of A). The post-run was 3 min. The flow rate was 1 mL/min, and the injection volume was 5 μL. The column thermostat was set to 30 °C, and the samples were kept at 4 °C in the sampler manager. The spectral characteristics of the monitored analytes were scanned in the wavelength range 210–400 nm, while detection wavelengths were set up at 320 nm (neochlorogenic acid, chlorogenic acid, trans-caffeic acid, trans-*p*-coumaric acid, trans-sinapic acid, trans-ferulic acid) and 372 nm (rutin, vitexin, quercetin, kaempferol). Compounds were identified and quantified by comparing the retention times of standard substances and comparing the run of the spectral UV lines of the analytes (Supplementary Figure S10). Data were collected and processed using Agilent Open Lab Chem Station software for LC 3D systems (Agilent Technologies).

### 3.4. Statistical Analysis

All analyses were performed in four repetitions. The Shapiro–Wilk test and the Kolmogorov–Smirnov test were performed to test the normality of all variables. All tested variables did not follow the normal distribution; therefore, Kruskal–Wallis and Wilcoxon tests were performed to find the significant differences between the tested variables. For a better understanding and graphical interpretation of the results, each cultivar was compared to the median value (horizontal line in graphs) using the Wilcoxon test. The Spearman correlation test at the significance level α = 0.05 was used to analyze the relationships between the variables. Descriptive statistics and normality tests were performed using MS Excel with the XLSTAT package [53]. Kruskal–Wallis and Wilcoxon tests were performed using RStudio software, version 1.2.5033 (RStudio Inc.: Boston, MA, USA) [54].

## 4. Conclusions

The main purpose of this investigation was to establish whether the concentration of flavonoids and main phenolic acids in flowers is connected with their concentration in grain and leaves. Such a relationship was not previously studied in buckwheat. According to the present results, the concentration of phenolic acids is independently determined in flowers, leaves, and grains. For buckwheat breeding purposes, it is essential to know whether the concentration of studied metabolites in different buckwheat plant parts is correlated or whether it is concentrated in diverse parts of plants differently due to a difference in genetic expression. This experiment shows the existence of diverse metabolite concentration patterns in flowers, leaves, and grains.

The highest concentration of chlorogenic acid and trans-sinapic acid was determined in flowers. For other studied phenolics, the highest concentration was established in the leaves, followed by flowers and then grains. Buckwheat flowers, up to now mostly neglected in the scientific literature, are an important source of diverse secondary metabolites, with a different pattern of their concentration compared to those of leaves or grains.

In chlorogenic acid, trans-caffeic acid, trans-sinapic acid, vitexin, and kaempferol, there were differences among cultivars. Significantly higher content of trans-caffeic acid was observed in Pyra compared to other cultivars. The cultivars Soldier Pond and FAG 120/80 contained the highest contents of trans-sinapic acid, while the Kasho-2 cultivar contained the highest content of chlorogenic acid. Higher values of kaempferol were established for Hrusowska and Soldier Pond compared to other cultivars. The highest content of vitexin among all cultivars was observed for Pyra. Among studied buckwheat cultivars, no significant differences in the contents of the three phenolic acids, trans-ferulic, trans-coumaric, and neochlorogenic acids were established.

The results confirmed the determining role of cultivar on the contents of chlorogenic acid, trans-caffeic acid, trans-sinapic acid, vitexin, and kaempferol in buckwheat plants. According to our results, the concentration of these phenolic acids is independently genetically determined in flowers, leaves, and grains.

It was found that several phenolic substances are synthesized and allocated in flowers, leaves, and grains independently. These differences should be considered when breeding buckwheat for the high concentration of selected phenolic substances and when selecting cultivars for diverse nutritional and pharmaceutical purposes.

## Figures and Tables

**Table 1:** Content of phenolic acids in leaves of investigated buckwheat cultivars (mg/g d.w., means ± standard deviations of four independent determinations).

Cultivar	Neochlorogenic Acid	Chlorogenic Acid	Trans-Caffeic Acid	Trans-Coumaric Acid	Trans-Sinapic Acid	Trans-Ferulic Acid
Pyra (*n* = 4)	0.666 ± 0.301	0.193 ± 0.003	0.102 ± 0.001	0.039 ± 0.001	ND	0.083 ± 0
Kasho-2 (*n* = 4)	0.849 ± 0	0.280 ± 0.003	0.026 ± 0	0.110 ± 0	ND	0.054 ± 0
Hrusowska (*n* = 4)	0.460 ± 0	0.222 ± 0	0.065 ± 0.005	0.164 ± 0	ND	0.079 ± 0
FAG 120/82 (*n* = 4)	0.665 ± 0.012	0.236 ± 0.001	0.014 ± 0	0.103 ± 0	0.008 ± 0	0.040 ± 0
Pulawska (*n* = 4)	0.310 ± 0.005	0.141 ± 0.006	0.019 ± 0	0.046 ± 0	ND	0.050 ± 0
Soldier Pond (*n* = 4)	0.411 ± 0.009	0.116 ± 0	ND	0.103 ± 0.001	0.011 ± 0.001	0.013 ± 0.003
Pennline 10 (*n* = 4)	0.661 ± 0.016	0.274 ± 0.017	0.049 ± 0	0.089 ± 0.005	ND	0.096 ± 0
Ballada (*n* = 4)	0.489 ± 0.011	0.201 ± 0.001	0.052 ± 0.001	0.057 ± 0.001	ND	0.060 ± 0.001

ND, not detected.

**Table 2:** Content of flavonoids in leaves of investigated buckwheat cultivars (mg/g d.w., means ± standard deviations of four independent determinations).

Cultivar	Rutin	Vitexin	Quercetin	Kaempferol
Pyra (*n* = 4)	28.211 ± 0.097	0.075 ± 0	0.461 ± 0.001	ND
Kasho-2 (*n* = 4)	31.069 ± 0.007	ND	0.127 ± 0	ND
Hrusowska (*n* = 4)	21.317 ± 0.021	0.050 ± 0	0.628 ± 0.002	0.018 ± 0.001
FAG 120/82 (*n* = 4)	22.901 ± 0.067	0.055 ± 0.001	0.345 ± 0.001	ND
Pulawska (*n* = 4)	17.742 ± 0.006	ND	0.234 ± 0	ND
Soldier Pond (*n* = 4)	29.281 ± 16.906	ND	0.914 ± 0.528	0.031 ± 0.001
Pennline 10 (*n* = 4)	29.665 ± 0.018	0.069 ± 0	0.664 ± 0.001	ND
Ballada (*n* = 4)	22.292 ± 12.87	0.031 ± 0	0.421 ± 0.001	ND

ND, not detected.

**Table 3:** Content of phenolic acids in flowers of investigated buckwheat cultivars (mg/g d.w., means ± standard deviations of four independent determinations).

Cultivar	Neochlorogenic Acid	Chlorogenic Acid	Trans-Caffeic Acid	Trans-Coumaric Acid	Trans-Sinapic Acid	Trans-Ferulic Acid
Pyra (*n* = 4)	0.221 ± 0.007	0.381 ± 0.007	0.057 ± 0.001	0.031 ± 0	0.015 ± 0	0.011 ± 0
Kasho-2 (*n* = 4)	0.190 ± 0.004	1.268 ± 0.004	0.047 ± 0	0.017 ± 0	0.015 ± 0	0.015 ± 0
Hrusowska (*n* = 4)	0.144 ± 0	0.275 ± 0.001	0.037 ± 0.001	0.014 ± 0	ND	0.007 ± 0
FAG 120/82 (*n* = 4)	0.200 ± 0.007	0.513 ± 0.002	0.053 ± 0.001	0.020 ± 0	0.023 ± 0	0.016 ± 0
Pulawska (*n* = 4)	0.121 ± 0.003	0.121 ± 0	0.010 ± 0	0.012 ± 0	ND	ND
Soldier Pond (*n* = 4)	0.188 ± 0	0.146 ± 0	ND	0.093 ± 0	0.028 ± 0.001	ND
Pennline 10 (*n* = 4)	0.124 ± 0.003	0.146 ± 0.01	0.025 ± 0	0.013 ± 0.004	ND	ND
Ballada (*n* = 4)	0.109 ± 0.489	0.209 ± 0.001	0.050 ± 0.001	0.011 ± 0	ND	ND

ND, not detected.

**Table 4:** Content of flavonoids in flowers of investigated buckwheat cultivars (mg/g d.w., means ± standard deviations of four independent determinations).

Cultivar	Rutin	Vitexin	Quercetin	Kaempferol
Pyra (*n* = 4)	12.756 ± 0.005	0.488 ± 0.001	0.161 ± 0	ND
Kasho-2 (*n* = 4)	21.255 ± 0.003	0.237 ± 0	0.260 ± 0.001	ND
Hrusowska (*n* = 4)	12.406 ± 0.006	0.314 ± 0.001	0.142 ± 0	ND
FAG 120/82 (*n* = 4)	18.917 ± 0.002	0.258 ± 0.005	0.215 ± 0	ND
Pulawska (*n* = 4)	7.410 ± 0.015	0.056 ± 0.293	0.089 ± 0	ND
Soldier Pond (*n* = 4)	20.847 ± 0.036	ND	1.680 ± 0.001	0.054 ± 0.001
Pennline 10 (*n* = 4)	9.264 ± 0.008	0.172 ± 0	0.122 ± 0	ND
Ballada (*n* = 4)	9.208 ± 5.316	0.354 ± 0.205	0.117 ± 0.068	ND

ND, not detected.

**Table 5:** Content of phenolic acids in grain of investigated buckwheat cultivars (mg/g d.w., means ± standard deviations of four independent determinations).

Cultivar	Neochlorogenic Acid	Chlorogenic Acid	Trans-Caffeic Acid	Trans-Coumaric Acid	Trans-Sinapic Acid	Trans-Ferulic Acid
Pyra (*n* = 4)	0.117 ± 0.002	0.075 ± 0.001	0.017 ± 0	0.011 ± 0.001	0.005 ± 0	0.004 ± 0
Kasho-2 (*n* = 4)	0.120 ± 0	0.087 ± 0	0.022 ± 0	0.012 ± 0	0.006 ± 0	0.003 ± 0
Hrusowska (*n* = 4)	0.111 ± 0.002	0.050 ± 0	0.012 ± 0	0.007 ± 0	0.008 ± 0	0.003 ± 0
FAG 120/82 (*n* = 4)	0.074 ± 0	0.047 ± 0.001	0.012 ± 0	0.009 ± 0	0.006 ± 0	0.003 ± 0
Pulawska (*n* = 4)	0.092 ± 0	0.027 ± 0	0.006 ± 0	0.006 ± 0.025	0.005 ± 0.025	ND
Soldier Pond (*n* = 4)	0.141 ± 0	0.090 ± 0.002	0.014 ± 0	0.013 ± 0.006	0.008 ± 0	0.003 ± 0
Pennline 10 (*n* = 4)	0.108 ± 0.001	0.054 ± 0	0.014 ± 0	0.007 ± 0	ND	ND
Ballada (*n* = 4)	0.216 ± 0.001	0.027 ± 0	ND	0.132 ± 0	0.011 ± 0	0.005 ± 0

ND, not detected.

**Table 6:** Content of flavonoids in grain of investigated buckwheat cultivars (mg/g d.w., means ± standard deviations of four independent determinations).

Cultivar	Rutin	Vitexin	Quercetin	Kaempferol
Pyra (*n* = 4)	4.017 ± 0.005	0.153 ± 0.001	0.036 ± 0	0.004 ± 0.001
Kasho-2 (*n* = 4)	5.900 ± 0.004	0.212 ± 0.1	0.055 ± 0	0.005 ± 0
Hrusowska (*n* = 4)	6.263 ± 0.001	0.063 ± 0	0.080 ± 0	0.011 ± 0.003
FAG 120/82 (*n* = 4)	5.019 ± 0.003	0.054 ± 0	0.052 ± 0	0.007 ± 0.001
Pulawska (*n* = 4)	2.791 ± 0.002	0.044 ± 0	0.036 ± 0	ND
Soldier Pond (*n* = 4)	7.510 ± 0.002	0.153 ± 0	0.062 ± 0	ND
Pennline 10 (*n* = 4)	4.069 ± 0	0.100 ± 0	0.034 ± 0	ND
Ballada (*n* = 4)	13.236 ± 0.009	0.010 ± 0	1.438 ± 0	0.063 ± 0

ND, not detected.

**Table 7:** The country of origin of the investigated buckwheat cultivars.

Cultivar Name	Country of Origin
Pyra	Czech Republic
Kasho-2	Japan
Hrusowska	Poland
FAG 120/82	Germany
Pulawska	Poland
Soldier Pond	USA
Pennline 10	USA
Ballada	Russia

## Data Availability

Supporting data are available in the supplement.

## Supplementary Materials

The following are available online at https://www.mdpi.com/article/10.3390/plants10061142/s1. Figure S1: Differences in the contents of trans-caffeic, trans-coumaric, trans-ferulic and trans-sinapic acids in leaves; Figure S2: Differences in the content of analysed flavonoids in leaves; Figure S3: Differences in the contents of trans-caffeic, trans-coumaric, trans-ferulic and trans-sinapic acids in flowers; Figure S4: Differences in the content of analysed flavonoids in flowers; Figure S5: Differences in the contents of trans-caffeic, trans-coumaric, trans-ferulic and trans-sinapic acids in groats; Figure S6: Differences in the content of analysed flavonoids in groats; Figure S7: Spearman correlation matrix displaying the relationships among all the tested acids and flavonoids; Figure S8: Dislocation of the tested phenolic acids in the different parts of plant; Figure S9: Dislocation of the tested flavonoids in the different parts of plant; Figure S10: Chromatograms of buckwheat leaves, flowers, grains and standards.
