# Supplementary material for: Concentrations of Phenolic Acids Are Differently Genetically Determined in Leaves, Flowers, and Grain of Common Buckwheat (Fagopyrum esculentum Moench)
Source: Plants (Basel). 2021 Jun 3;10(6):1142. doi: 10.3390/plants10061142 (PMC8228752; doi:10.3390/plants10061142)
Supplement: Supplementary file 1 [file plants-10-01142-s001.zip › plants-1243149-supplementary.pdf]

plants

MDPI

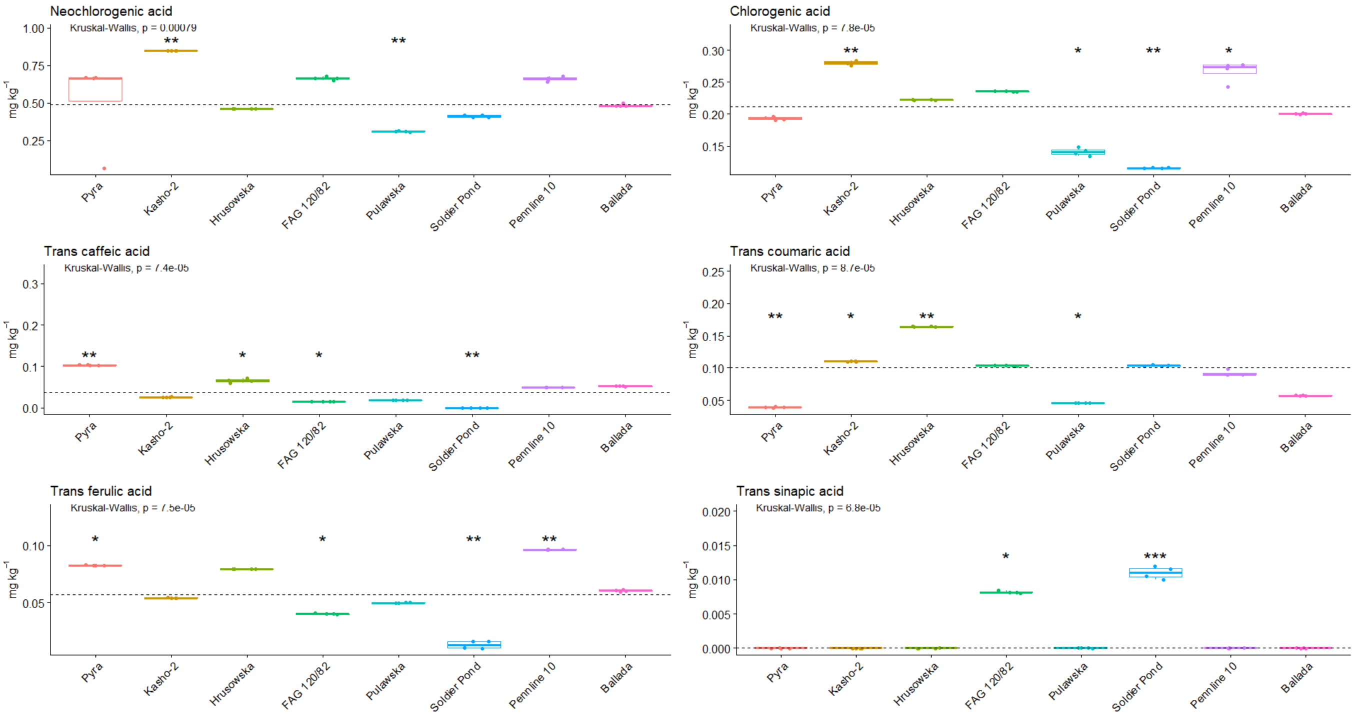

Figure S1. Differences in the contents of trans-caffeic, trans-coumaric, trans-ferulic and trans-sinapic acids in leaves. The symbols: * ($p < 0.05$), ** ($p < 0.01$), *** p ($p < 0.001$) represent a statistically significant differences compared to the median value.

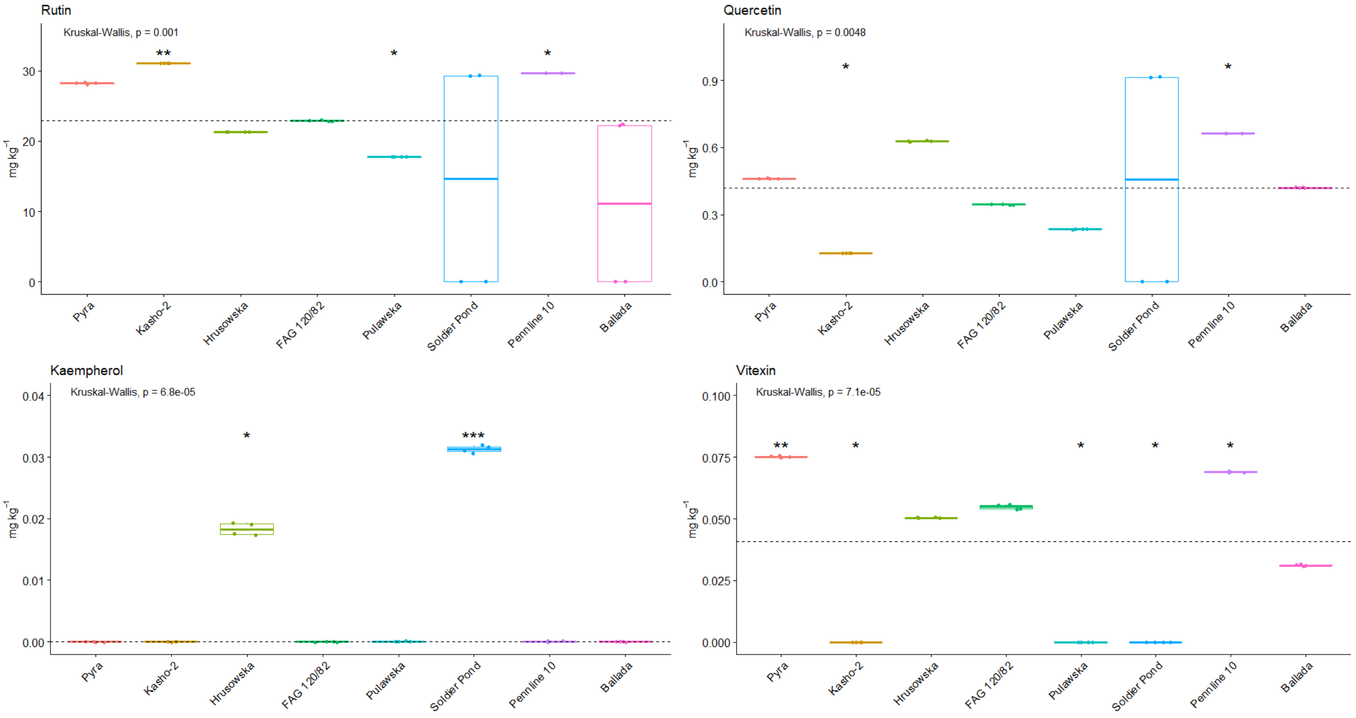

Figure S2. Differences in the content of analysed flavonoids in leaves. The symbols: * ($p < 0.05$), ** ($p < 0.01$), *** p ($p < 0.001$) represent a statistically significant differences compared to the median value.

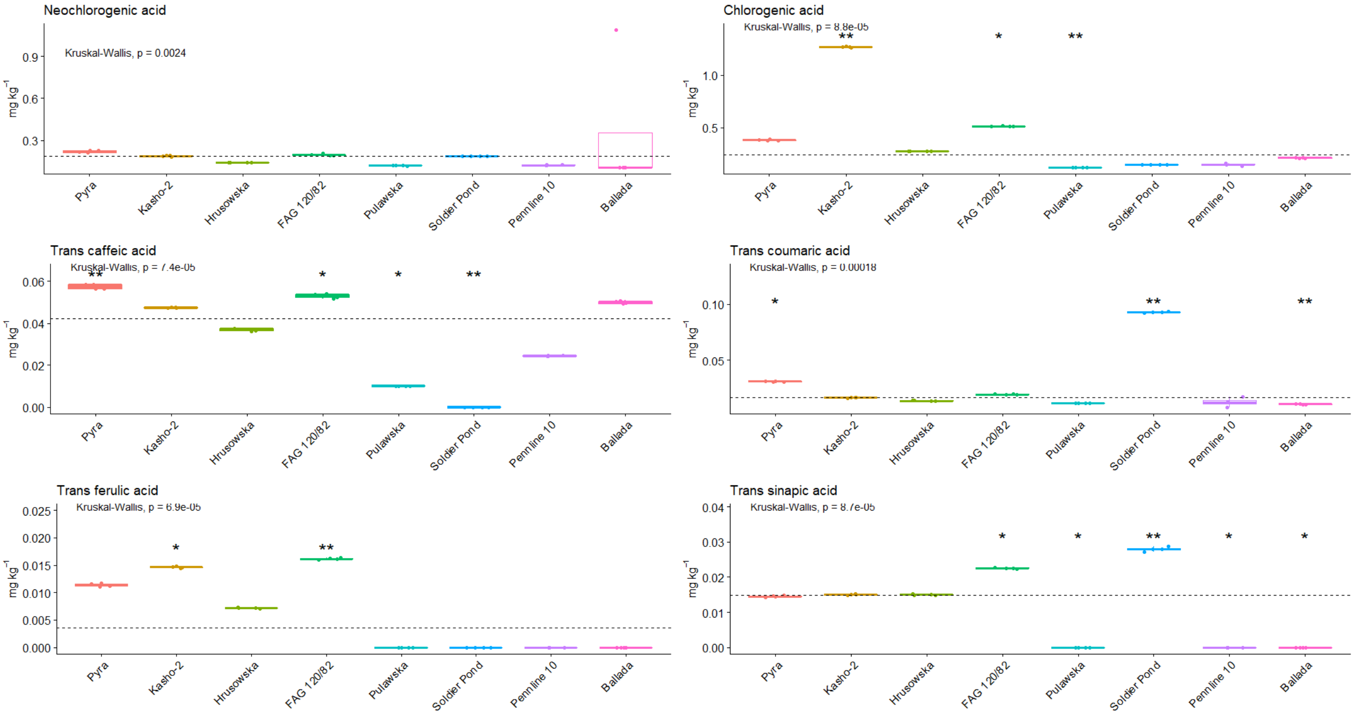

Figure S3. Differences in the contents of trans-caffeic, trans-coumaric, trans-ferulic and trans-sinapic acids in flowers. The symbols: * ($p < 0.05$), ** ($p < 0.01$), *** p ($p < 0.001$) represent a statistically significant differences compared to the median value.

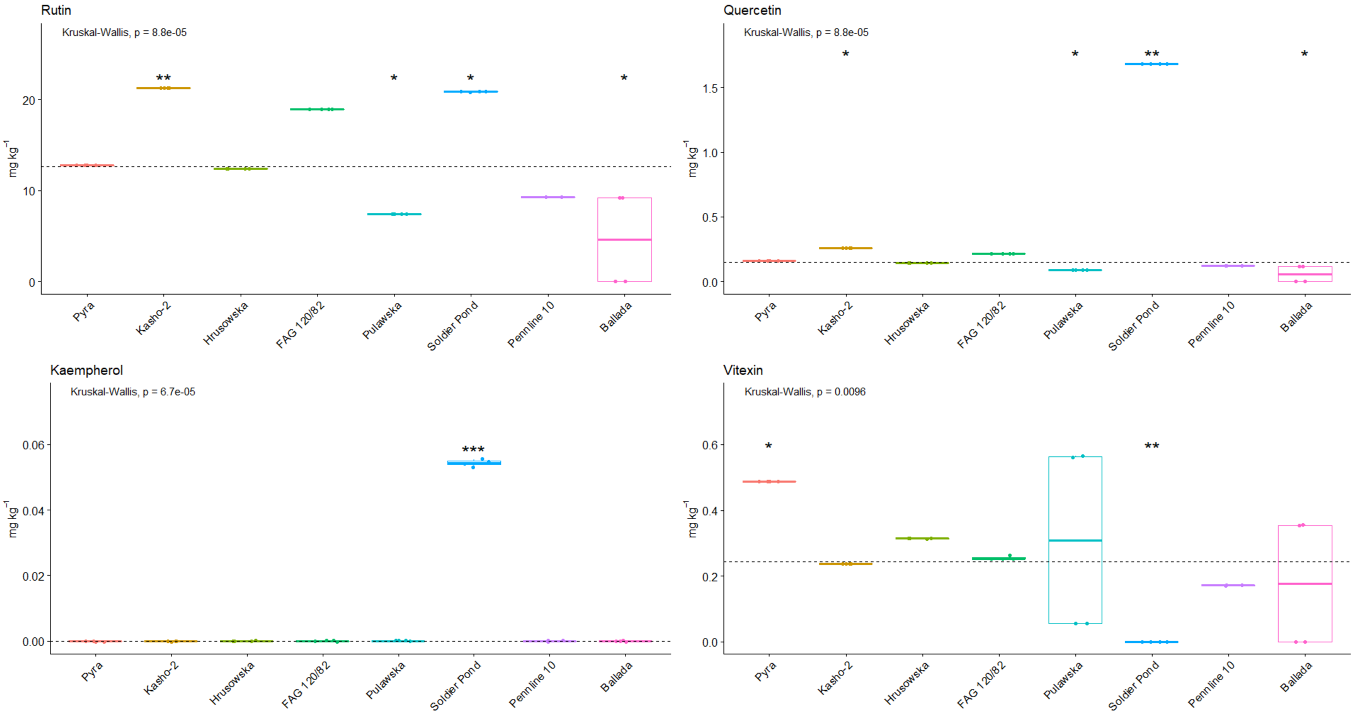

Figure S4. Differences in the content of analysed flavonoids in flowers. The symbols: * ($p < 0.05$), ** ($p < 0.01$), *** p ($p < 0.001$) represent a statistically significant differences compared to the median value.

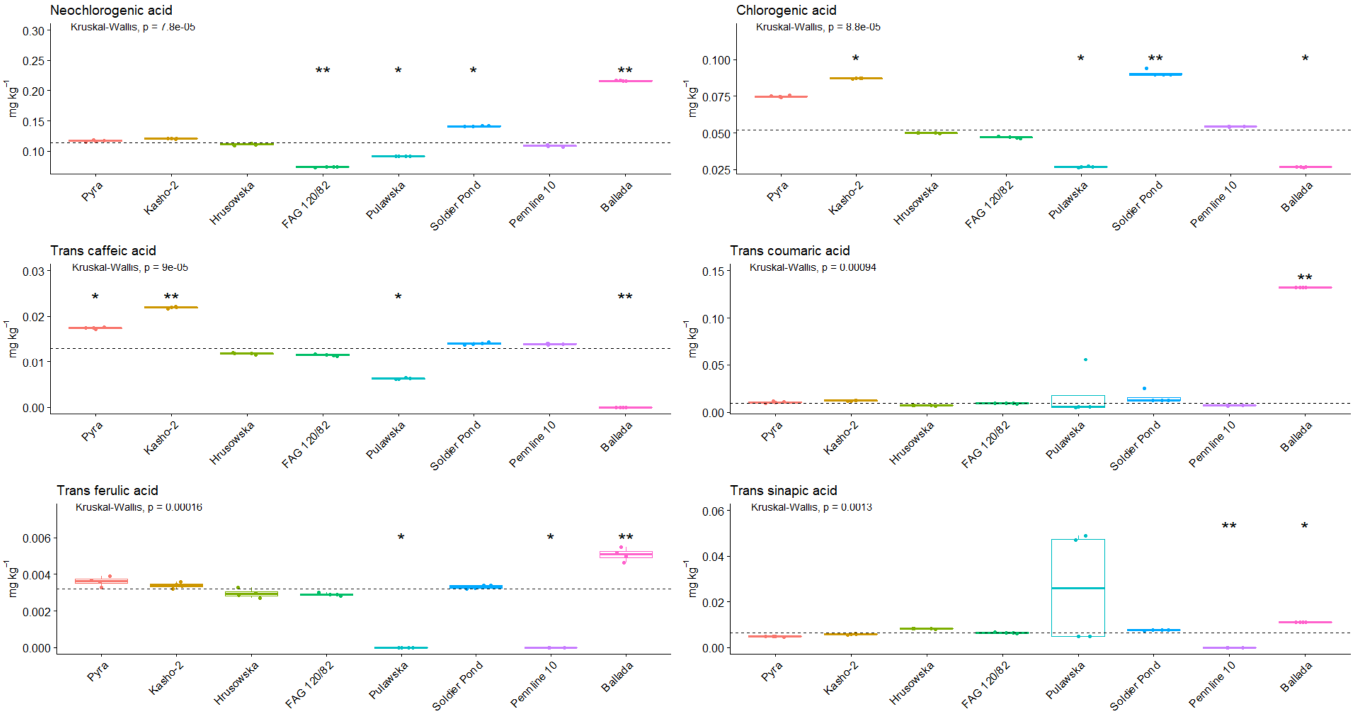

Figure S5. Differences in the contents of trans-caffeic, trans-coumaric, trans-ferulic and trans-sinapic acids in groats. The symbols: * ($p < 0.05$), ** ($p < 0.01$), *** p ($p < 0.001$) represent a statistically significant differences compared to the median value.

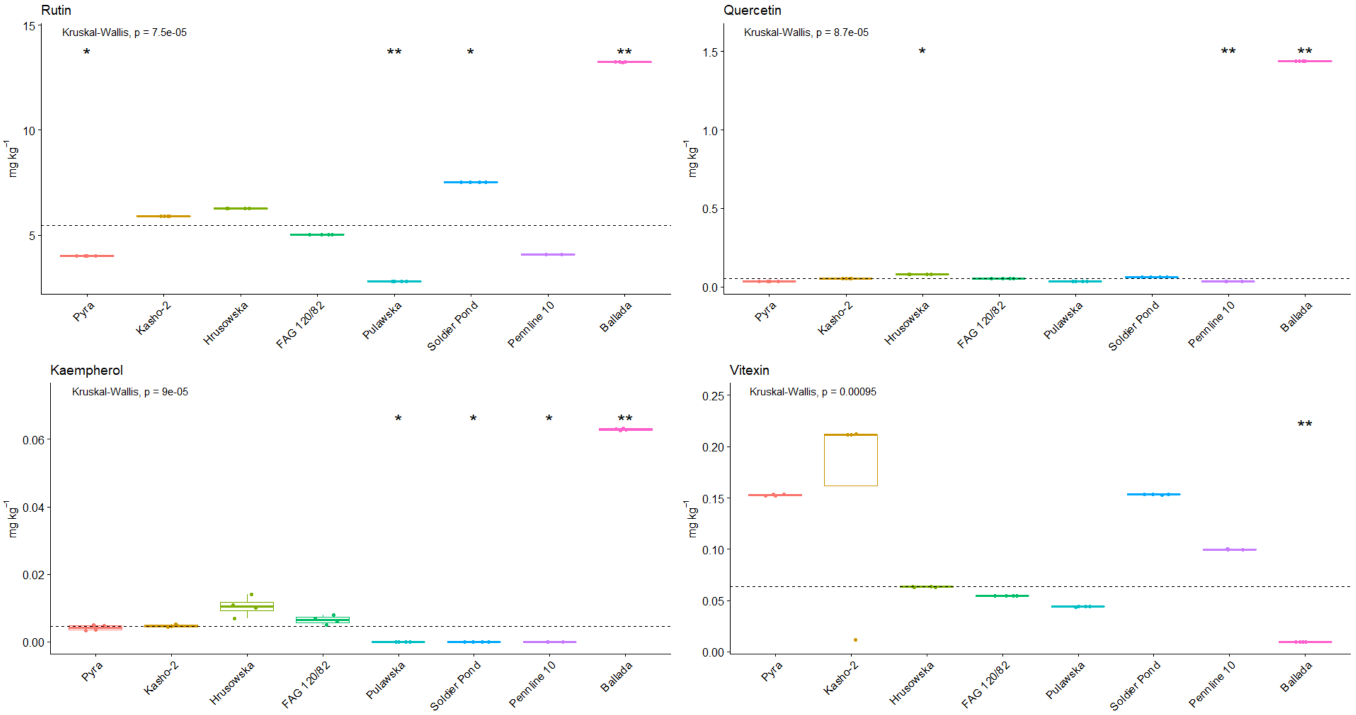

Figure S6. Differences in the content of analysed flavonoids in groats. The symbols: * ($p < 0.05$), ** ($p < 0.01$), *** p ($p < 0.001$) represent a statistically significant differences compared to the median value.

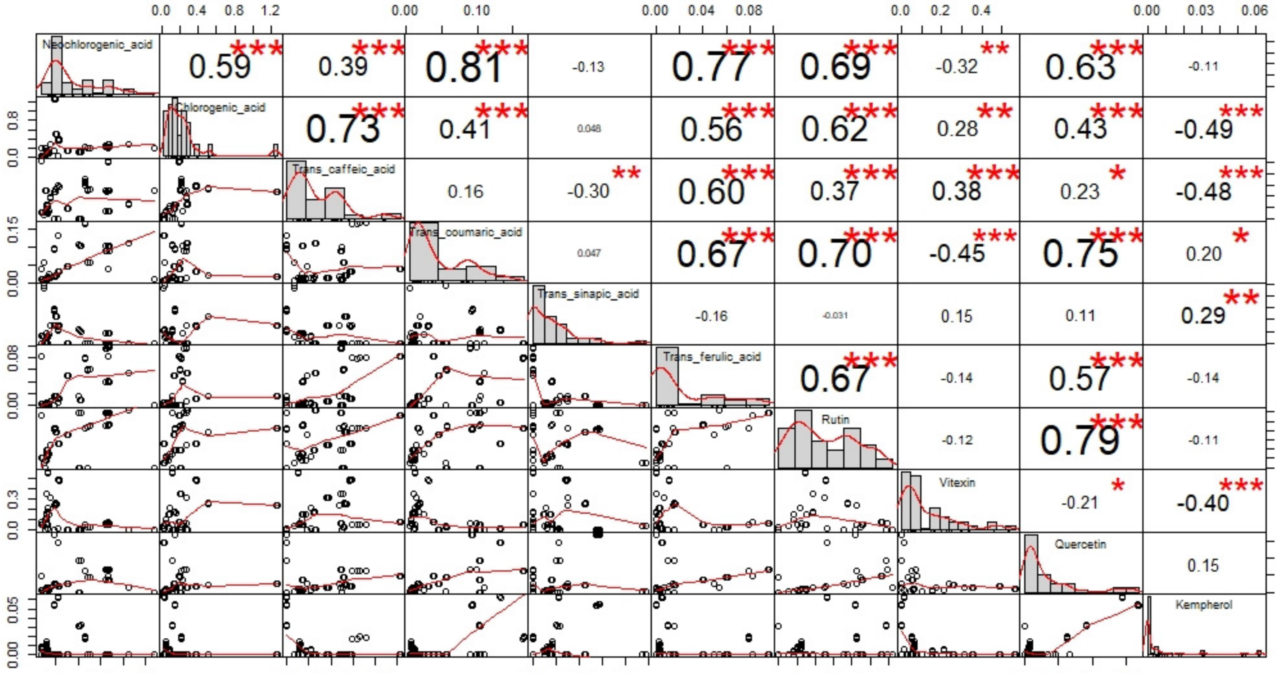

Figure S7. Spearman correlation matrix displaying the relationships among all the tested acids and flavonoids. Statistically significant differences between the tested variables are labeled as follows: * (p < 0.05), ** (p < 0.01), *** p (p < 0.001).

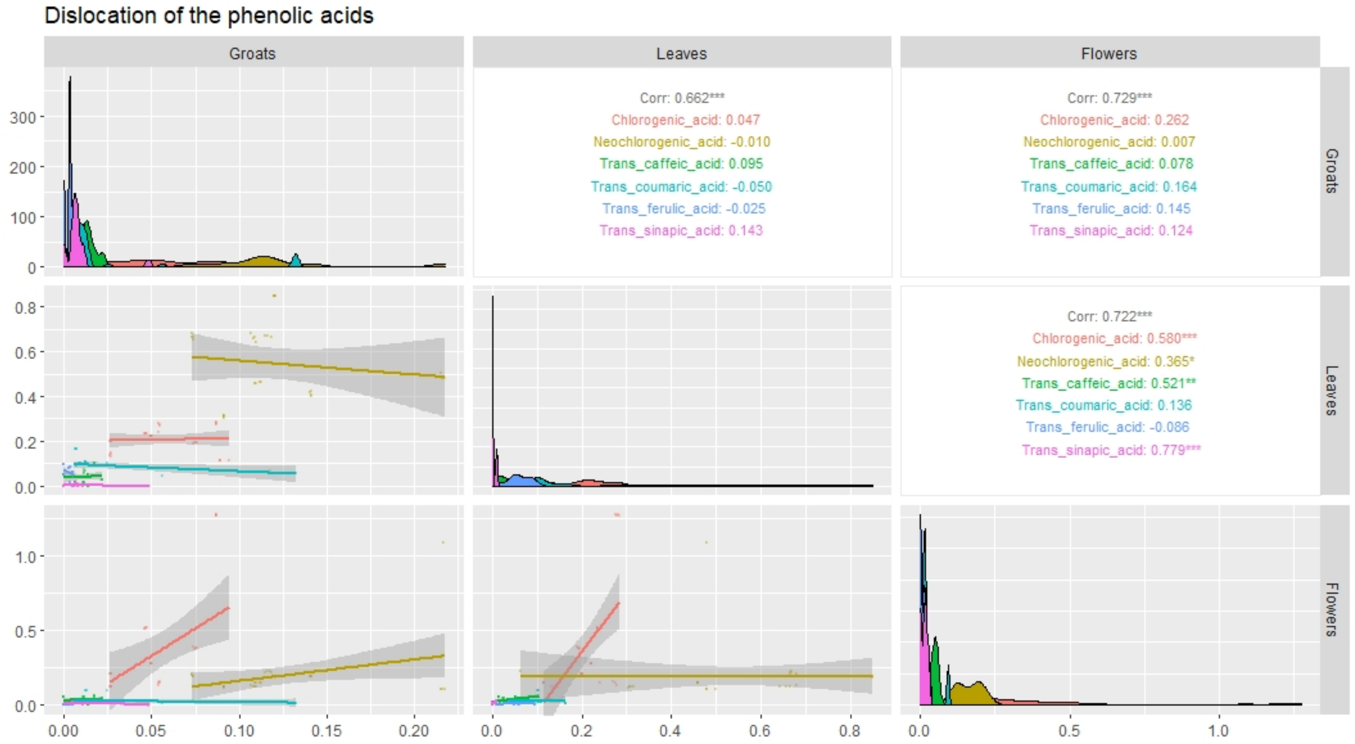

Figure S8. Dislocation of the tested phenolic acids in the different parts of plant. Statistically significant differences between the tested acids in different organs of the plant are labeled as follows: * (p < 0.05), ** (p < 0.01), *** p (p < 0.001).

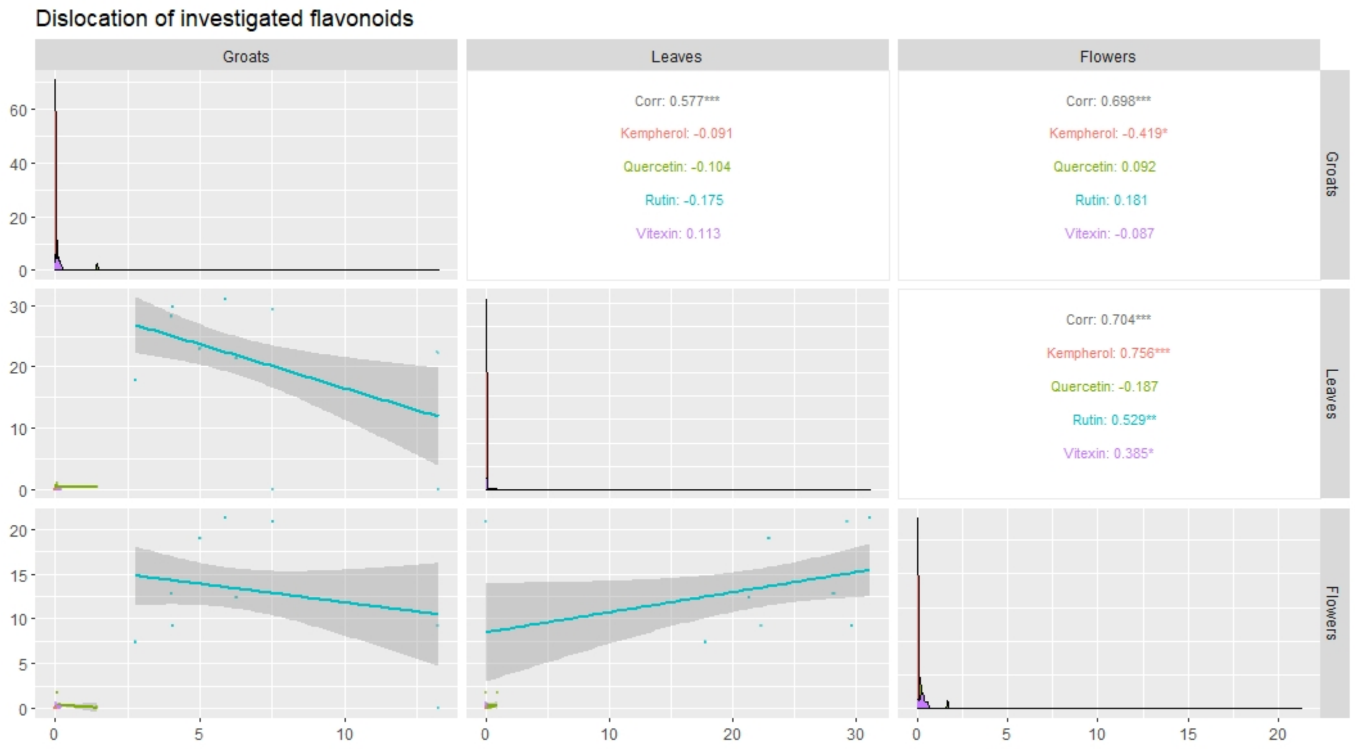

Figure S9. Dislocation of the tested flavonoids in the different parts of plant. Statistically significant differences between the tested flavonoids in different organs of the plant are labeled as follows: * ($p < 0.05$), ** ($p < 0.01$), *** p ($p < 0.001$).

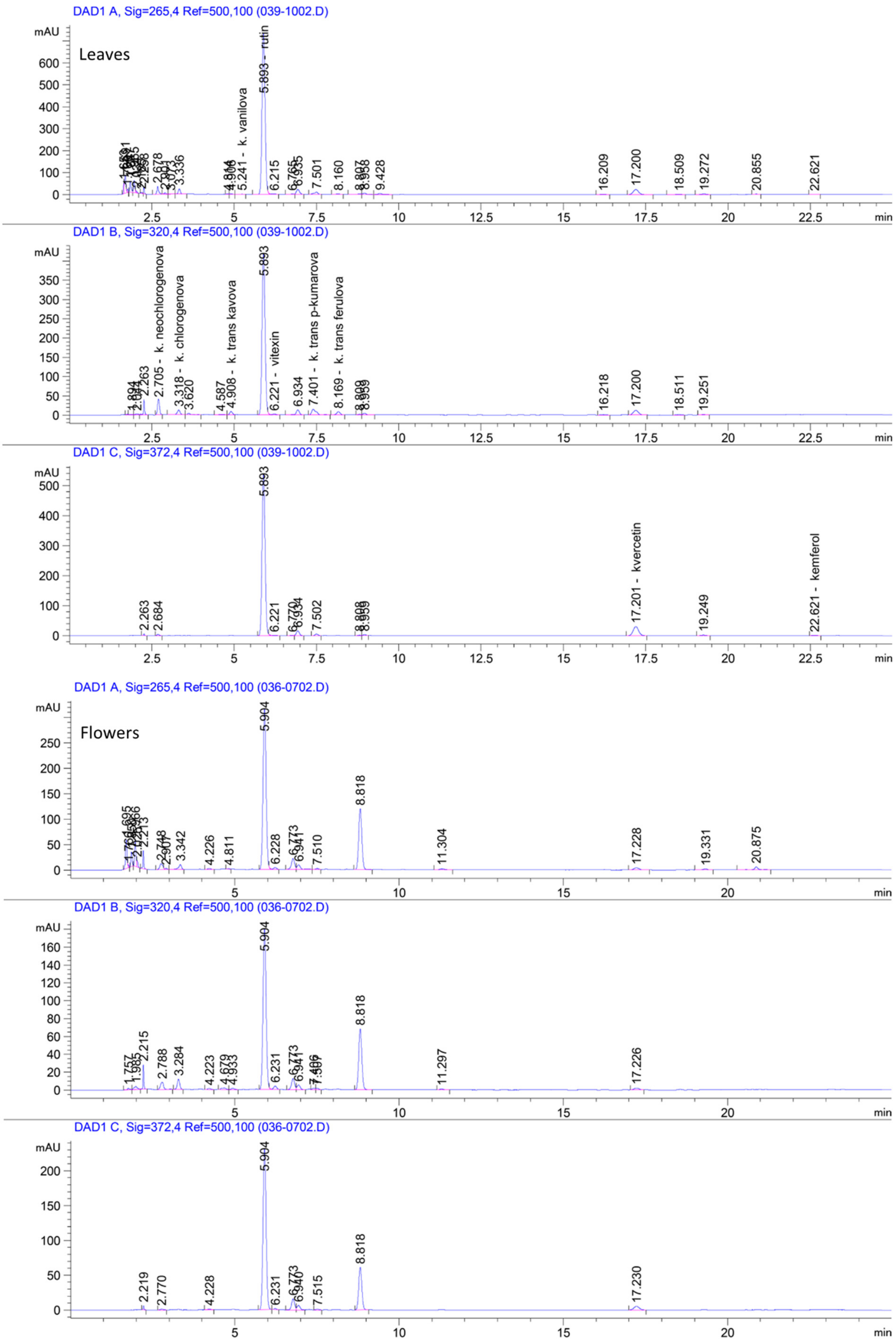
DAD1 A, Sig=265,4 Ref=500,100 (039-1002.D)
Leaves
mAU
5.893 - rutin
5.241 - k. vanilova
6.215
6.935
7.501
8.160
9.428
16.209
17.200
18.509
19.272
20.855
22.621
3.336
min
DAD1 B, Sig=320,4 Ref=500,100 (039-1002.D)
2.263
2.705 - k. neochlorogenova
3.318 - k. chlorogenova
3.620
4.587
4.908 - k. trans kavova
5.893
6.221 - vitexin
6.934
7.401 - k. trans p-kumarova
8.169 - k. trans ferulova
16.218
17.200
18.511
19.251
DAD1 C, Sig=372,4 Ref=500,100 (039-1002.D)
2.263
2.684
5.893
6.221
7.502
17.201 - kvercetin
19.249
22.621 - kemferol
DAD1 A, Sig=265,4 Ref=500,100 (036-0702.D)
Flowers
5.904
8.818
11.304
17.228
19.331
20.875
2.213
3.342
4.226
4.811
6.228
6.773
7.510
DAD1 B, Sig=320,4 Ref=500,100 (036-0702.D)
2.215
2.788
3.284
4.223
4.933
5.904
6.231
6.773
8.818
11.297
17.226
DAD1 C, Sig=372,4 Ref=500,100 (036-0702.D)
2.219
2.770
4.228
5.904
6.231
7.515
8.818
17.230

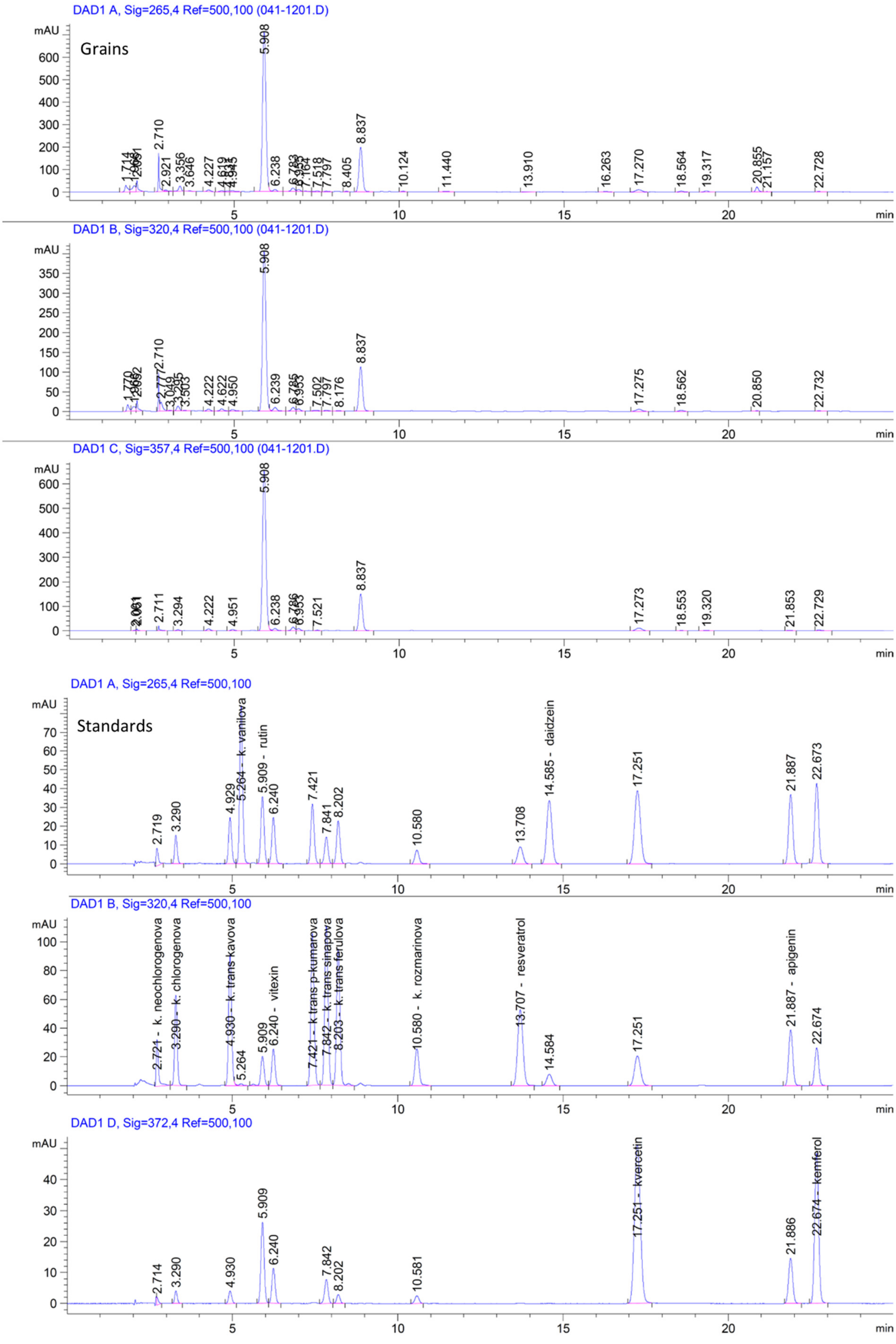

Figure S10. Chromatograms of buckwheat leaves, flowers, grains and standards.
